# Social Media Use and Serious Psychological Distress Among Adolescents

**DOI:** 10.2196/57041

**Published:** 2024-05-23

**Authors:** Riti Shimkhada, Ninez A Ponce

**Affiliations:** 1 Center for Health Policy Research, University of California, Los Angeles, CA, United States; 2 Health Policy and Management, Fielding School of Public Health, University of California, Los Angeles, CA, United States

**Keywords:** social media, socials, youth, adolescents, teens, teenager, mental health, mental illness, mental disease, mental illnesses, psychological distress, psychological, psychology

## Abstract

This Research Letter describes the increasing trend of almost-constant social media use among California adolescents and the association with serious psychological distress, focusing on the influence of familial and experiential factors.

## Introduction

Increasing recognition of the potential dangers of excessive social media use on mental health has led to numerous calls for restraint and associated legal cases, as highlighted by the recent advisory issued by the US Surgeon General [1-3]; however, these dangers remain understudied. We examined social media use trends between 2019 and 2021 among adolescents in California. We further examined the association between almost-constant social media use and psychological distress using 2021 data, controlling for familial dynamics and adverse childhood experiences (ACEs), which are predictors of mental health [4] but have largely been overlooked in previous studies.

## Methods

### Survey and Data Collection

We used the California Health Interview Survey (CHIS) 2019-2021 data sets [5] for adolescents aged 12-17 years to examine trends in social media use. For a regression analysis examining the association between almost-constant social media use and psychological distress among adolescents, we used the 2021 data set with responses from 24,453 households, including 1169 participating adolescents. The CHIS randomly selects one adult to interview in each randomly sampled household, wherein households are selected using a geographically stratified address-based sample design. Following parental permission, surveys are conducted directly with a randomly sampled adolescent in the household. The survey, conducted either on the web or by telephone, includes a comprehensive set of health-related questions. Respondents are asked to report on typical daily use of social media on the following scale: less than a few times a day, a few times a day, many times a day, and almost constantly or more. We categorized this scale into a two-category variable wherein the category “almost constantly or more” is referred to as almost-constant social media use. Psychological distress was measured using the Kessler 6 series [6]; for this analysis, we examined the category of likely having had serious psychological distress in the past year. Family connection was measured through a series of questions, including how often the adolescent felt they were able to talk to family about their feelings, how often they felt family stood by them during difficult times, how often they felt safe and protected by the adult at home, and how often they had at least two nonparent adults taking a genuine interest in them. Adolescents responding “little to never” to any of these questions were assigned to a group characterized by little to no family connection. Adolescents responding having had at least one ACE were assigned “yes” to the ACEs variable.

### Statistical Analysis

Demographic variables, including age, gender, race and ethnicity, and socioeconomic status, were included in the analysis as covariates. The data were weighted to account for the complex survey design and to generate population-level estimates. We used multivariate logistic regression models to examine the association between almost-constant social media use and serious psychological distress, controlling for demographic characteristics, family factors, and ACEs. All analyses were conducted using STATA 16.1.

### Ethical Considerations

This study used deidentified, publicly available data, which does not constitute human subjects research as defined by regulation 45 CFR 46.102 of the US Department of Health and Human Services [7], and thus does not require ethics board approval. All CHIS respondents receive an initial survey invitation letter with a US $2 preincentive. Participation is voluntary and all participants provide informed consent before participating in the survey.

## Results

Almost-constant social media use for the youngest teens (aged 12-14 years) increased significantly between 2019 and 2021, whereas this increase was not noted for older teens (aged 15-17 years). Among 12-14–year-old females, the rate of almost-constant use was 18.1% (95% CI 11.2-25.0) in 2019, 22.2% (95% CI 15.4-29.1) in 2020, and 28.9% (95% CI 20.8-37.0) in 2021; among 15-17–year-old females, the rate of almost-constant use was 33.0% (95% CI 25.8-40.2) in 2019, 26.9% (95% CI 20.8-32.9) in 2020, and 29.1% (95% CI 20.8-37.3) in 2021. Among male adolescents, almost-constant social media use increased significantly between 2019 and 2021 for both age groups. Among 12-14–year-old males, the rates were 11.9% (95% CI 6.2-17.6) in 2019, 22.7% (95% CI 16.4-28.9) in 2020, and 23.7% (95% CI 18.4-29.0) in 2021; among 15-17–year-old males, the rates were 14.1% (95% CI 9.8-18.4) in 2019, 22.4% (95% CI 17.3-27.6) in 2020, and 28.9% (95% CI 22.9-34.9) in 2021. By 2021, there were no longer significant differences in almost-constant social media use according to age group.

The rates of almost-constant social media use were the highest for teens living in poverty, those who have experienced ACEs, those who reported little to no family connection, and those who reported serious psychological distress (Table 1).

Regression analyses suggested a significant positive association between almost-constant social media use and psychological distress controlling for ACEs, family connection, and demographics (Table 2).

**Table 1.** Association of characteristics of adolescents (12-17 years old) with almost-constant social media use from the California Health Interview Survey 2021 (N=1169).

Characteristic		Almost-constant social media use, % (95% CI)
Total (all adolescents)		27.62 (24.19-31.35)
**Age (years)**		
	12-14	26.23 (21.48-31.61)
	15-17	28.98 (24.30-34.16)
**Gender^a^**		
	Female	28.97 (23.61-35.00)
	Male	26.35 (22.40-30.72)
**Federal Poverty Level (%)**		
	<300	33.19 (27.65-39.23)
	≥300	22.61 (19.26-26.35)
**Race** ^**b**^		
	Asian	28.66 (21.02-37.76)
	Latino	30.23 (24.95-36.09)
	Black, African American, and other	22.72 (12.81-37.04)
	Two or more	27.94 (17.60-41.31)
	White	23.17 (18.21-29.01)
**Serious psychological distress**		
	Yes	37.36 (31.09-44.09)
	No	22.63 (19.27-26.38)
**Adverse childhood experiences**		
	Yes	36.17 (30.72-42.01)
	No	20.95 (17.48-24.91)
**Family connection**		
	Little to none	34.02 (28.47-40.04)
	More than little	22.21 (18.42-26.52)

a While the California Health Interview Survey collects data on nonbinary gender, for sample size considerations, we report only male and female categories here.

b The sample size was too small to produce reliable estimates for the Black/African American population alone, American Indian or Alaska Native, and Native Hawaiian or Pacific Islander populations.

**Table 2.** Association between almost-constant social media use and serious psychological distress among adolescents (aged 12-17 years) from the California Health Interview Survey 2021 (N=1169).

Variable		Adjusted odds ratio (95% CI)	*P* value
**Social media use per day**			.003
	Almost constant	1.673 (1.204-2.323)	
	Less than almost constant	reference	
**Age (years)**			.003
	12-14	reference	
	15-17	1.710 (1.205-2.429)	
**Gender^a^**			.005
	Female	1.597 (1.155-2.209)	
	Male	reference	
**Federal Poverty Level (%)**			.92
	<300	1.017 (0.709-1.460)	
	≥300	reference	
**Race** ^**b**^			
	Asian	0.641 (0.377-1.089)	.10
	Latino	0.674 (0.469-0.967)	.03
	Black, African American, and other	0.807 (0.357-1.824)	.60
	Two or more	1.133 (0.617-2.079)	.68
	White	reference	
**Adverse childhood experiences**			<.001
	Yes	2.403 (1.736-3.327)	
	No	reference	
**Family connection**			<.001
	Little to none	2.171 (1.502-3.139)	
	More than little	reference	

a While the California Health Interview Survey collects data on nonbinary gender, for sample size considerations, we report only male and female categories here.

b The sample size was too small to produce reliable estimates for the Black/African American population alone, American Indian or Alaska Native, and Native Hawaiian or Pacific Islander populations.

## Discussion

Our work is the first to examine the association between social media use and psychological distress controlling for ACEs and the adolescent-reported level of family connection, both of which are significantly associated with psychological distress [4]. Even when controlling for these and other demographic variables, almost-constant social media use remained significantly associated with psychological distress. Our key limitation is the cross-sectional nature of the data. Other limitations include the analysis of self-reported data on social media use, which may be subject to recall or social desirability bias, and possible other confounders not included in the analysis. Our work is consistent with prior research that finds increasing trends in use of social media among the youngest teens [8] and potential adverse mental health impacts from high or almost-constant social media use [9-12].

## Abbreviations

ACE: adverse childhood experience
CHIS: California Health Interview Survey
